# Osteogenic Differentiation Potential of Mesenchymal Stem Cells Using Single Cell Multiomic Analysis

**DOI:** 10.3390/genes14101871

**Published:** 2023-09-26

**Authors:** Duojiao Chen, Sheng Liu, Xiaona Chu, Jill Reiter, Hongyu Gao, Patrick McGuire, Xuhong Yu, Xiaoling Xuei, Yichen Liu, Jun Wan, Fang Fang, Yunlong Liu, Yue Wang

**Affiliations:** Department of Medical and Molecular Genetics, Indiana University School of Medicine, Indianapolis, IN 46202, USA

**Keywords:** mesenchymal stem cells, single-cell multiome, osteogenic differentiation

## Abstract

Mesenchymal stem cells (MSC) are multipotent stem cells that can differentiate into multiple cell types, including osteoblasts, chondrocytes, and adipocytes. Osteoblast differentiation is reduced during osteoporosis development, resulting in reduced bone formation. Further, MSC isolated from different donors possess distinct osteogenic capacity. In this study, we used single-cell multiomic analysis to profile the transcriptome and epigenome of MSC from four healthy donors. Data were obtained from ~1300 to 1600 cells for each donor. These cells were clustered into four groups, indicating that MSC from different donors have distinct chromatin accessible regulatory elements for regulating gene expression. To investigate the mechanism by which MSC undergo osteogenic differentiation, we used the chromatin accessibility data from the single-cell multiome data to identify individual-specific enhancer–promoter pairs and evaluated the expression levels and activities of the transcriptional regulators. The MSC from four donors showed distinct differentiation potential into osteoblasts. MSC of donor 1 showed the largest average motif activities, indicating that MSC from donor 1 was most likely to differentiate into osteoblasts. The results of our validation experiments were consistent with the bioinformatics prediction. We also tested the enrichment of genome-wide association study (GWAS) signals of several musculoskeletal disease traits in the patient-specific chromatin accessible regions identified in the single-cell multiome data, including osteoporosis, osteopenia, and osteoarthritis. We found that osteoarthritis-associated variants were only enriched in the regions identified from donor 4. In contrast, osteoporosis and osteopenia variants were enriched in regions from donor 1 and least enriched in donor 4. Since osteoporosis and osteopenia are related to the density of bone cells, the enrichment of variants from these traits should be correlated with the osteogenic potential of MSC. In summary, this study provides large-scale data to link regulatory elements with their target genes to study the regulatory relationships during the differentiation of mesenchymal stem cells and provide a deeper insight into the gene regulatory mechanism.

## 1. Introduction

Single-cell sequencing technologies have been widely used in the detection of multiple omics data at the single-cell level. It provides a deeper characterization of cellular diversity and states [1] and allows understanding cellular heterogeneity, discovering novel cell types and biomarkers for clinical applications [2]. To understand how gene regulatory programs are established and how cell states are specified, transcriptomic and epigenetic measurements have been integrated to provide new insight into biological processes [3]. The Chromium Single Cell Multiome technology + Gene Expression developed by 10x Genomics (Pleasanton, CA, USA) can jointly profile the accessible regions and the gene expression within the same cell simultaneously at the single-cell resolution. With a unified view of a cell’s open chromatin landscape and gene expression profile, it is possible to resolve how cell types and states are established, discover new gene regulatory interactions, and interpret epigenetic profiles with key expression markers.

Mesenchymal stem cells (MSC) are multipotent cells having the capability to self-renew and differentiate into different cell types under defined conditions [4]. There are multiple transcription factors that can affect the differentiation progresses through regulating the expression of specific genes [5]. Previous studies mainly focused on either the identification of transcription factors or differentially expressed genes during the differentiation progress [6,7,8,9]. However, the regulatory relationship between those key transcription factors and genes requires deeper investigation.

In this study, using the MSC derived from four donors, we profiled the transcriptome and epigenome of the mesenchymal stem cells simultaneously. Single-cell multiome technology provided a more confident way to link the regulatory elements with gene expression to explore the gene regulatory mechanism during differentiation. We used the single-cell multiome data to identify the co-accessible regulatory elements and predict the regulation relationships between genes and the key transcription factors involved in osteogenic differentiation. These analysis results would help to elucidate the regulatory mechanism of MSC differentiation in osteoblast cells.

## 2. Materials and Methods

### 2.1. Cell Preparation

Poietics™ Normal Human Bone Marrow Derived Mesenchymal Stem Cells (hMSC) were obtained from the bone marrow of healthy adult volunteers through bilateral punctures of the posterior iliac crests, as performed by Lonza. Four lots of Poietics™ hMSC, each from a different donor, were purchased from Lonza. Demographic information is listed in Table S1. MSCBMT™ Basal Media (PT-3238) and MSCGM™ SingleQuots Supplement Kit (PT-4105) were purchased from Lonza to ensure the cells’ health in culture. The media were changed every 3 days. The materials were used according to Lonza’s limited use license. The cells tested CD105+, CD166+, CD29+, CD44+, CD14-, CD34-, CD45- by flow cytometry. The cells tested positive for adipogenic lineage, chondrogenic lineage, and osteogenic lineage using in vitro assays.

### 2.2. Sequencing Library Construction

The single cell multiome analysis was conducted using a 10X Chromium single cell system (10x Genomics, Inc. Pleasanton, CA, USA) and a NovaSeq 6000 sequencer (Illumina, Inc., San Diego, CA, USA). After the cell monolayer reached 70–80% confluency, the MSC from each of the four donors were collected and combined in equal numbers to create a mixed cell preparation. One million of the MSC mixed cells were centrifuged at 300× *g* for 5 min to remove culture media. The final single cell suspension was washed 3 times with PBS buffer without calcium and magnesium, plus 0.4% BSA. The single-cell suspension was then assessed for cell viability and quantity by using a hemocytometer under a microscope. Cells were then lysed with 0.1× lysis buffer for 4 min to isolate nuclei, based on the protocol of Demonstrated Protocol Nuclei Isolation Complex Sample ATAC GEX Sequencing, CG000375 RevB (10x Genomics, Inc. Pleasanton, CA, USA). A final nuclei concentration of 3000/µL was used for targeted cell recovery of 10,000 cells. Following the Chromium NextGEM Multiome ATAC GEX User Guide, CG000338_RevB (10x Genomics, Inc. Pleasanton, CA, USA), tagmentation of nuclei preparation was first performed. Thereafter, briefly, along with the single cell multiome gel beads and partition oil, the single nuclei master mixture containing tagmented single nuclei suspension was transferred onto a Next GEM Chip J, and the was chip loaded to the Chromium Controller for GEM generation and barcoding, followed by pre-amplification PCR, ATAC library preparation, cDNA synthesis, and cDNA library preparation. At each step, the quality of cDNA, ATAC library, and cDNA library was examined by Bioanalyzer. The resulting ATAC and cDNA libraries were sequenced separately, cDNA library for 28 bp and 91 bp and ATAC library for 50 bp paired-end sequencing on Illumina NovaSeq 6000.

### 2.3. Data Analysis Procedure

These RNA+ATAC multiome data were firstly demultiplexed using genotype-free pipeline from souporcell [10] to separate the cell barcodes from different individuals. The 10x Genomics multiome RNA + ATAC data were processed using Seurat [11] and Signac [12]. RNA-seq data were processed following the standard workflow. Low-quality droplets were defined as those containing < 1200 total unique molecular identifiers, or the percentage of mitochondrial reads was >15%. We used SCTransform to normalize RNA counts, and we used principal component analysis (PCA) to reduce the dimensionality of RNA. ATAC-seq peaks were identified for each individual separately using MACS2 [13] and the CallPeaks function in Signac with the argument additional.args = ‘–max-gap 50’. The FeatureMatrix function was used to quantify the fragment counts for each peak per cell. TSSEnrichment and NucleosomeSignal functions were used to compute the per-cell quality. Cells with nucleosome signal score > 2 and Transcript Start Site (TSS) enrichment score < 1.5 were removed. Term Frequency-Inverse Document Frequeny (TF-IDF) was used to normalize ATAC peaks and latent semantic indexing (LSI) to reduce the dimensionality of ATAC data. Finally, 1–50 PCA dimensions and 2–50 LSI dimensions were used to construct the weighted nearest neighbor (WNN) graph for clustering.

### 2.4. Motif Activity

Motif analysis was conducted following the suggested workflow in Signac. We used chromVAR [14] and JASPAR 2018 database [15,16] to identify transcription factor motifs within the accessible chromatin regions of each cell. A merged peak by cell binary matrix was constructed and the addGCBias function was used to correct for GC bias based on BSgenome.Hsapiens.UCSC.hg38. For each transcription factor motif, we calculated the bias-corrected z-scores for each cell. For each individual, we calculated the average transcription factor motif enrichment z-score for cells within the same individual.

### 2.5. Differential Analysis for Identifying Subject-Specific Key Regulators

For each individual, subject-specific transcriptional regulators were selected by jointly analyzing their gene expression levels and motif activities. The R package Presto was used to evaluate the ability of each gene or motif to separate cell groups by calculating a *p*-value using the Wilcox rank-sum test and the area under curve (‘AUC’) statistics. We used the Beniamini–Hochberg method to correct the *p*-values. Subject-specific key regulators were defined as the transcription factors with significantly differentially expressed genes and significantly different motif activities as compared to the cells of other individuals.

### 2.6. Detect the Co-Accessible Regions

For the single-cell ATAC data, we used Cicero [17] to identify the enhancers and promoters with coordinated accessibility. Chromatin regions that appeared in less than 1% of cells in all individuals were removed. We first identified all the interacted elements within 500,000 bp using run cicero function from Cicero. All the pairs with negative correlation were removed. Promoters were defined as the regions 1500 bp upstream or 500 bp downstream from the TSS of annotated genes. Cicero identified all correlated chromatin regions and we only considered the correlated enhancer–promoter pairs. We also extracted the *p*-value for the correlation between the accessibility signal and used the Beniamini–Hochberg method to correct the *p*-values. Only significantly correlated enhancer and promoter pairs with false discoverary rate adjusted *p* value (FDR) < 0.05 were used in the following analysis.

### 2.7. Infer Gene Regulatory Networks

Due to high heterogeneity across individuals and relative homogeneity within individuals, we used a pseudobulk approach to minimize false positive identification. Previous reports [18] suggest that single-cell differential expression (DE) methods can be biased and are prone to false discovery. In contrast, pseudobulk methods outperform generic and specialized single-cell DE methods. We used PECA (paired expression and chromatin accessibility) [19] to infer the regulatory networks that lead to the subject-specific gene expression profile. Pseudobulk RNA-seq and ATAC-seq for each individual were generated by summing the sequencing reads of all the cells with the subject barcode. The profiles of paired pseudobulk RNA-seq and ATAC-seq were used as input of PECA, where target gene expressions were modeled as a function of expression of transcription factor (TF) and activities of regulatory elements (RE). The activities of the RE were based on gene expression of recruited chromatin regulators (CR) and RE’s openness. The recruitment of a CR to an RE was predicted utilizing the TF binding to the RE and CRs interacting with the TFs. For each of the four subjects, PECA was used to model how each known cis-acting regulatory element may interact with relevant transcriptional regulators to affect the expression of its target genes from the resultant pseodobulk RNA and ATAC-seq data. The annotations of putative enhancer–promoter pairs, which are required by PECA as input, were acquired from the co-accessibility analysis from Section 2.6. Only enhancer–promoter pairs with a correlation coefficient larger than 0.3 were included in the analysis.

### 2.8. GO Analysis and KEGG Pathway Analysis

GO analysis was conducted using ‘clusterProfiler’ [20]. We extracted the genes from the TF–TG relationships predicted by PECA and used these gene sets to identify the enriched gene ontology terms or signaling/metabolic pathways.

### 2.9. GWAS Signal Enrichment with Single-Cell Genomic Annotations

For subject-specific open chromatin regions identified from pseudobulk ATAC-seq data, we examined heritability based on the GWAS data of several traits related to bone diseases, including osteoporosis [21], osteopenia [22], and osteoarthritis [23], using polyTest [24]. We followed the detailed methods mentioned in the paper published by Chiou, J., et al. (2021) [25] to calculate the enrichment score for each trait.

### 2.10. Experimental Validation for Osteogenic Differentiation

The Mesenchymal Stem Cell Osteogenesis Kit (Millipore-Sigma, St. Louis, MO, USA) was utilized to induce the differentiation of MSC into osteocytes. Osteogenesis differentiation media were prepared according to the instruction of the kit. The MSC were cultured in Falcon^®^ 8-well glass culture slides, and, once the cell monolayer had reached full confluency, the differentiation media were added into the chambers. Following four weeks of cultivation, the osteogenic differentiation status of the four MSC samples was assessed using ScienCell™’s Alizarin Red S (ARS) staining quantification assay (ARed-Q). This approach provided a precise and sensitive means of semi-quantifying ARS in a monolayer of stained cells. In order to compare the staining outcomes of the four MSC samples, we employed a Python program. Initially, the program reads each image and extracts the CIELAB [26] color of each pixel. Next, it determines the similarity of each pixel’s color to pure red by computing the ΔE* CIE76 version [27] of the distance between the two colors. Finally, the program generates a box plot illustrating the values of the distances to pure red.

## 3. Results

### 3.1. Identification of Enhancer and Promoter Regions with Single-Cell Multiome Data

**Figure 1:**
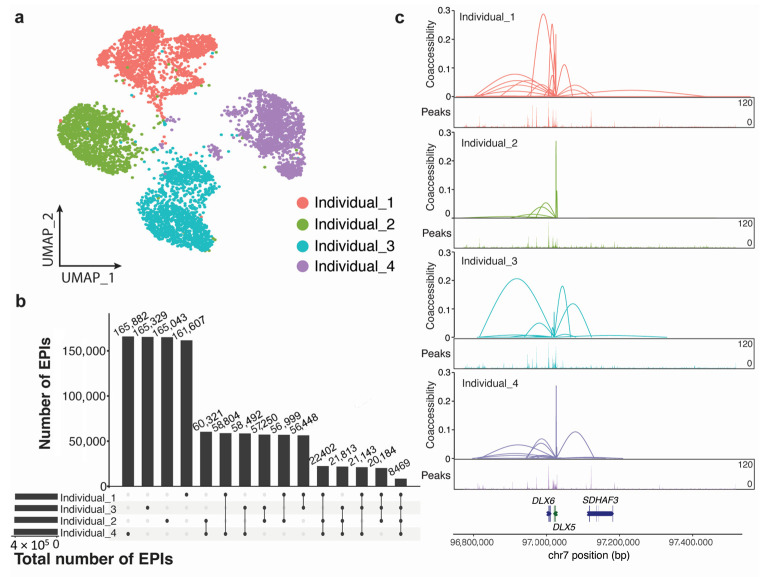
Identification of co-accessible regulatory elements. (**a**) UMAP plot of MSC of four donors. Each cell cluster represented MSC in one individual; (**b**) Upset plot of numbers of co-accessed enhancers and promoters; (**c**) Co-accessed enhancers with the promoter of *DLX5* in each donor.

We performed single-cell multiome sequencing on MSC obtained from four donors. Single-cell suspensions were obtained from each donor and then combined in equal numbers. Taking the advantage of the 10x Chromium Next GEM Single Cell Multiome ATAC + Gene Expression sequencing technology, we profiled gene expression and chromatin accessibility simultaneously. We collected 1635, 1342, 1496, and 1356 cells from each donor, respectively. The MSC were divided into four distinct groups, each consisting of a comparable number of cells and exhibiting clear separation between them (Figure 1a). As the cells are derived from the same primary cell type but obtained from various donors, we do not expect much heterogeneity among the cells from the same donor. Therefore, as demonstrated in the UMAP, the primary source of variation lies in the differences between the donors. There are some subclusters in the same donor, suggesting there might be potential differences among the subclusters. The difference is smaller than the donor difference, which might be trivial.

To study the regulatory mechanism in MSC, we first defined the regulatory regions. Promoter regions were defined as from 1500 bp upstream to 500 bp downstream from the transcription start sites of genes. Enhancers were identified as regions with significant correlation with promoters using Cicero [17] (corr_fdr < 0.05). We observed that these correlated enhancer–promoter pairs demonstrated an individual-specific pattern (Figure 1b). For example, different individuals showed different numbers of enhancers interacting with the promoter of *DLX5* (Figure 1c). In general, MSC of each donor showed distinct accessible regulatory elements to regulate gene expression.

### 3.2. Common Regulatory Networks across Individuals Demonstrated the Stemness Function of MSC

**Figure 2:**
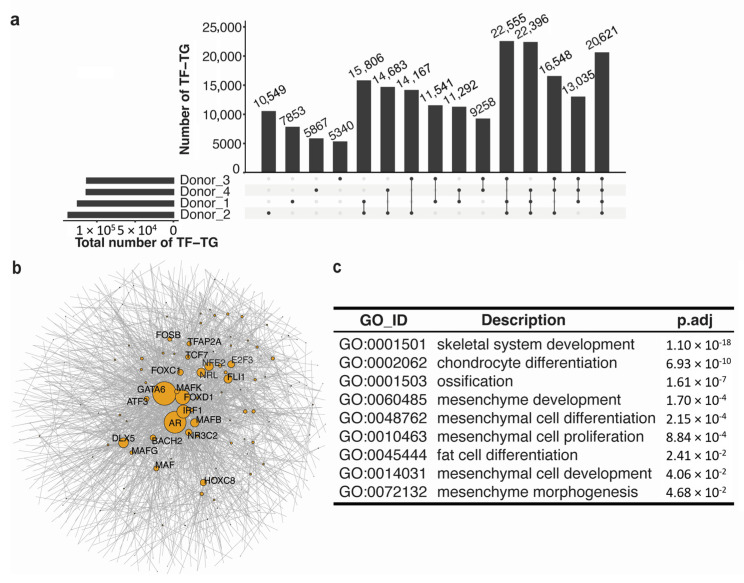
Common regulatory network across individuals. (**a**) Upset plot of numbers of TF–TG relationships predicted by PECA using paired gene expression and regulatory regions; (**b**) Network graph of common TF–TG relationships across individuals with corr(TF, TG) > 0.7 or corr(TF, TG) < −0.7. The colored nodes represent transcription factors, and the size of the circle is proportional to the number of genes targeted by each TF; (**c**) GO analysis result of target genes in common regulatory networks.

To understand the role of regulatory elements in the differentiation progress of mesenchymal stem cells, we used PECA [19] to infer the relationships between genes and transcription factors from the paired gene expression and chromatin accessibility of regulatory elements. We observed 20,621 TF–TG (target gene) relationships shared across all the four donors, and 5340 to 10,549 TF–TG relationships were specific to one donor (Figure 2a). We constructed the regulation network for the common TF–TG relationships and identified several transcription factors that are reported to be involved in the differentiation progress of MSC (Figure 2b). For instance, *GATA6* is known to regulate smooth muscle cell differentiation progress [28], and DLX5 is the major regulator driving the differentiation of MSC into the osteoblasts [29]. To further investigate the function of genes in the TF–TG relationships that were identified in all four donors, we conducted gene ontology enrichment analysis for all the genes detected in the common regulatory networks. Our analysis showed that these genes were enriched in functions associated with both stem cell differentiation (e.g., chondrocyte and adipocyte differentiation) and the stemness function of MSC (e.g., proliferation and development) (Figure 2c).

### 3.3. Individual-Specific Regulatory Networks Demonstrated the Differentiation Progress of MSC

**Figure 3:**
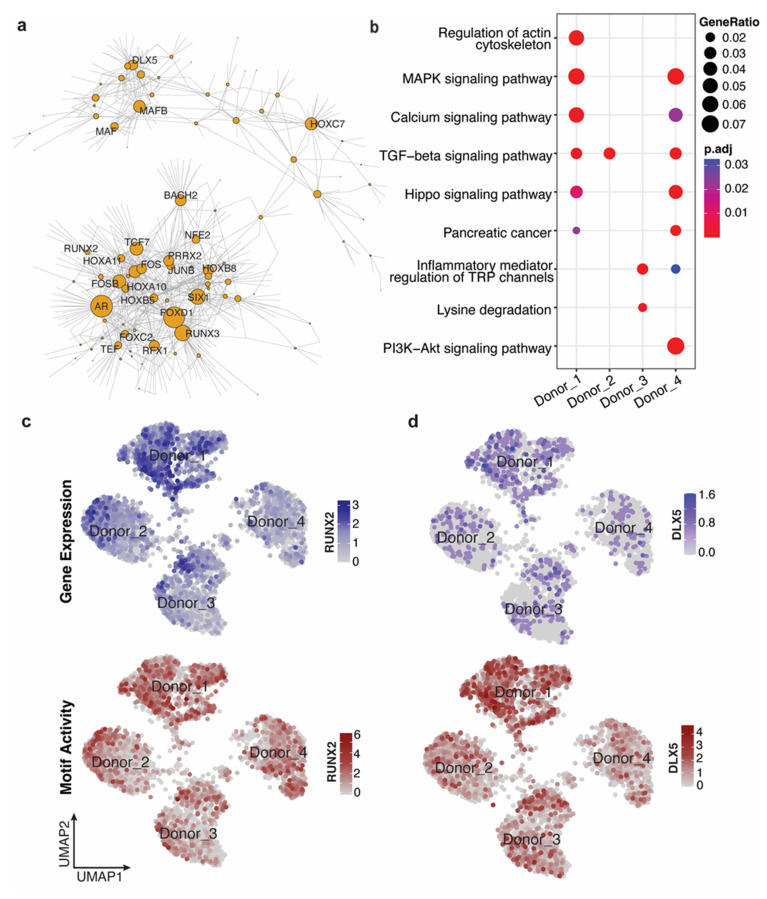
Individual-specific regulatory networks demonstrated the differentiation progress. (**a**) Regulatory network of MSC from donor 1 identified by PECA; (**b**) Dotplot of KEGG pathway analysis for target genes of *RUNX2*, *DLX5*, *HOXB7*, *FOXC2*, and *HOXA2*; (**c**,**d**) UMAP plots of gene expression distribution and motif activities distribution of *RUNX2* and *DLX5*.

Besides the common TF–TG relationships across all individuals indicating the stemness function of MSC, we also observed thousands of TF–TG relationships that are specific to one donor. To investigate their unique characteristics, we conducted function analysis on the genes in the regulatory networks in one individual whose TFs demonstrated higher gene expression and motif activities compared with the other three individuals. We observed transcription factors reported to be involved in the differentiation of several cell types across these donor-specific networks (Figure 3a and Figure S1). Within donor 1’s specific network, we found *RUNX2* and *DLX5*, which play key regulatory roles in osteogenic differentiation, and we found *FOXC2*, *HOXB7*, *HOXA2*, known as having osteogenic differentiation regulation function (Figure 3a, Table S2) [29]. In addition, signaling pathway analysis for the genes in the TF–TG relationships specific to donor 1 demonstrated the enrichment of MAPK signaling pathway and TGF-β signaling pathway (Figure 3b); both pathways were reported to play significant roles in controlling the osteogenic differentiation progress [30].

To identify the differentially active motifs between individuals, we computed a per-cell motif activity for each known motif in JASPAR using chromVAR [14]. We further compared the gene expression level and the motif activities for the key regulators in donor 1 with the other donors (Figure 3c,d, Table S3). *RUNX2* and *DLX5* both showed significantly higher gene expression levels (*RUNX2*: FDR < 3.07 × 10^−199^; *DLX5*: FDR < 1.11 × 10^−25^) and motif activities levels (*RUNX2*: FDR < 2.95 × 10^−77^; *DLX5*: FDR < 1.36 × 10^−117^) in donor 1 (Figure 3c,d). In general, our analysis demonstrated that MSC from donor 1 showed a clear tendency of osteogenic differentiation.

### 3.4. MSC from Different Donors Showed Varied Osteogenic Differentiation Potentials

**Figure 4:**
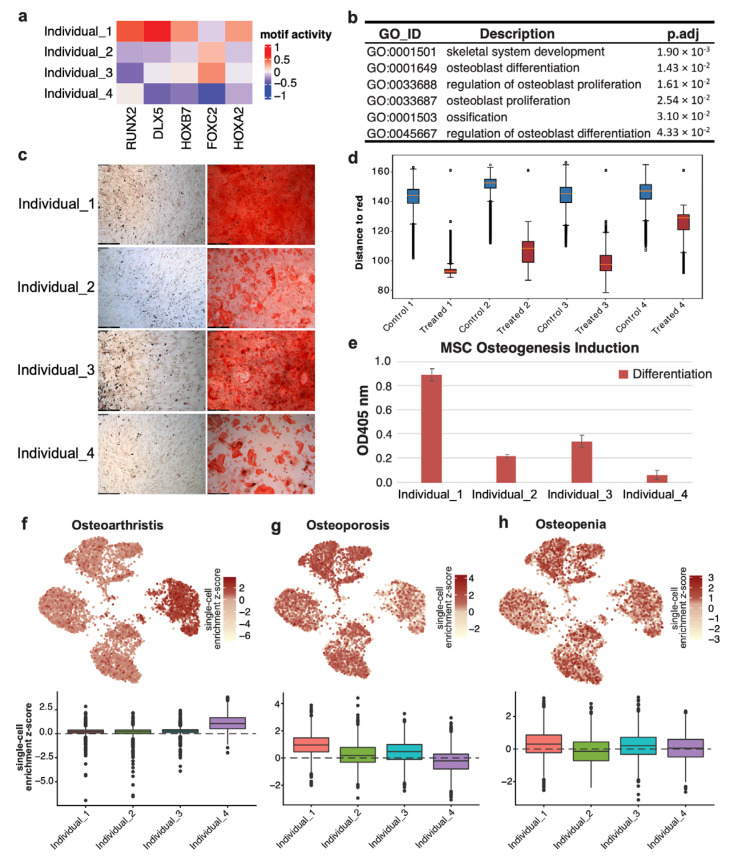
Heterogeneity osteogenic differentiation potential and enrichment for bone-related genome-wide association study (GWAS) signal across donors. (**a**) Heatmap of average motif activities of key regulators involved in the osteogenic differentiation; (**b**) Gene ontology (GO) analysis results for targeting genes of key regulators involved in the osteogenic differentiation in donor 1 specific networks; (**c**) Validation experiment results for osteogenic differentiation potential across donors. Differentiated cells were stained for calcification. Images are representative of stained cells. The total magnification of a low power objective lens combined with a 10× eyepiece lens is 100× magnification; (**d**) The calcification (Alizarin Red) staining signal of the four MSC samples under the control condition and being induced with osteogenic differentiation medium. The shorter the distance to red, the stronger the staining signal; (**e**) Alizarin Red Staining (ARS) half quantification assay results for the validation; (**f**–**h**) UMAP plots and boxplots of the single-cell enrichment z-scores for osteoarthritis, osteoporosis, and osteopenia.

We assessed the osteogenic differentiation potential of mesenchymal stem cells obtained from four donors by analyzing the motif activities of transcription factors known to be involved in osteogenic differentiation, namely *RUNX2*, *DLX5*, *HOXB7*, *FOXC2*, and *HOXA2* [5,29,30]. We observed that cells from donor 1 showed the highest average motif activities, while donor 4 showed the lowest motif activities (Figure 4a), indicating that MSC in donor 1 was most likely to differentiate into osteoblasts. We then checked the function of the target genes of these transcription factors in the individual-specific networks. We observed the gene ontology terms related to the differentiation and the proliferation of osteoblasts only in donor 1 (Figure 4b), indicating that these transcription factors can contribute to the osteogenic differentiation progress.

We performed experimental validation using MSC obtained from these donors to confirm our findings. The results of osteogenic staining showed that cells from donor 1 exhibited a greater level of osteogenic differentiation (Figure 4c). Our half quantification assay results also confirmed that cells from donor 1 had the highest osteogenic activity among the four donors, while cells from donor 4 had the lowest osteogenic activity. These findings were consistent with the motif activity predicted from our single-cell data (Figure 4d,e).

### 3.5. Heterogeneity Enrichment for Bone-Related Genome-Wide Association Studies Signal across Individuals

Based on the observation and validated differences in osteogenic differentiation among donors, we hypothesized that these donors may exhibit varying levels of susceptibility to bone-related diseases. To address the variability in genetic association enrichment among donors, we examined the enrichment of GWAS signals in accessible regions associated with osteoporosis [21], osteopenia [22], and osteoarthritis [23] (Figure 4f–h). We only observed the enrichment of variants associated with osteoarthritis in donor 4 (Figure 4f). We also observed variability in the enrichment of variants associated with bone density-related traits, osteoporosis, and its hallmark osteopenia, in accessible regions among individuals. The highest enrichment of these variants was found in donor 1, while the lowest one was found in donor 4 (Figure 4g,h). Given that osteoporosis and osteopenia are linked to bone cell density, the enrichment of variants associated with these traits should be correlated with the osteogenic potential of MSC. Our findings showed that the enrichment of GWAS signals from these two traits correlated with the motif activities of the key transcription factors that regulate osteogenic differentiation, as determined by our analysis of single-cell multiome data.

## 4. Discussion

In this study, we simultaneously profiled the transcriptome and epigenome of mesenchymal stem cells at the single cell level, providing a more comprehensive understanding of the underlying gene regulatory mechanism. Our analysis of the single-cell multiome data allowed us to explore the co-accessible regulatory elements and their corresponding target gene expression levels. Our findings indicate that common regulatory networks among donors were related to the stemness function of the mesenchymal stem cells, while donor-specific ones were related to differentiation into specific cell types. The results further suggest that regulatory networks in cells with a higher potential for osteogenic differentiation may be associated with bone density-related diseases.

In this study, we utilized single-cell multiome data to investigate the differentiation of MSC. Unlike the single molecular assay, which can only measure one aspect of the cell state, single-cell multiome sequencing technology is capable of measuring both transcriptome and epigenome simultaneously to avoid the computational challenge of accurately integrated data from two separate assays and the bias that is tissue dependent [31]. By combining cutting-edge bioinformatics analysis methods, single-cell multiome data can be utilized to infer the cell-type specific chromatin interaction. Such results compliment with the other experimental approaches, such as HiC or single-cell level (single-cell HiC [32]), with much less complicated experimental procedures [33,34]. The paired gene expression and chromatin accessibility data generated by single-cell multiome also provided a unique opportunity to identify direct target genes of a list of putative transcriptional regulators, which allows functional inference of regulatory networks.

Our work shows that single-cell multiome data can provide unique information to reveal the heterogeneity in the regulatory landscape, which determines cell fate even before changes occur in the transcriptome. We can also utilize single-cell multiome data to identify the interacted promoters and enhancers and link these regulator elements with their target genes. For the multipotent MSC, which have the capability of differentiating into multiple cell types, transcription factors binding in the regulatory elements combined with the gene expression profile can explore the gene regulatory interactions driving the differentiation. The results of this analysis will aid in the comprehension of the regulatory mechanisms involved in biological processes in mesenchymal stem cells. A particular subset of bone marrow stromal cells (BMSCs) is believed to function as skeletal stem cells [35,36], capable of differentiating into osteoblast, chondrocyte, and adipocyte. It has been reported that *RUNX2* and *DLX5* play crucial roles in osteogenesis of skeletal cells, which aligns with our findings in MSCs. *ZEB2* has been predicted as a specific osteogenic regulator in skeletal stem cells.

While here we focus on heterogeneity among donors, a further study on the gene expression, chromatin status, and relevant gene regulatory network of markers associated with osteoporosis, osteopenia, and osteoarthritis will help us understand the potential driving force for the disease progression and potential targets for therapeutic use.

One of the next steps in our analysis is to validate the presence of other well-known networks associated with osteogenesis, such as *MAPK* and *COLL1*. Further insights into the functional characteristics of these networks can be gained by performing knockdown experiments on key regulator genes and examining the resultant changes in genes, gene ontology, and pathways. It is important to note that our current study is based on data from only four donors. Expanding our dataset to include more individuals will increase our confidence in the validity of our discoveries. Consequently, a future study will focus on collecting data from a larger pool of donors.

## Data Availability

Sequence data are available in Gene Expression Omnibus (GEO) with accession number GSE.228669.

## Supplementary Materials

The following supporting information can be downloaded at: https://www.mdpi.com/article/10.3390/genes14101871/s1, Figure S1: Donor-specific gene regulatory network. (**a**–**c**) Gene regulatory networks predicted by PECA in donors 2, 3, and 4 with corr(TF, TG) > 0.5 or corr(TF, TG) < −0.5; Table S1: Demographic information of the four donors. Table S2: Donor-specific network predicted by PECA. Tab 1, Donor 1 specific network; Tab 2, Donor 2 specific network; Tab 3, Donor 3 specific network; Tab 4, Donor 4 specific network. Table S3: Key regulators identified for each donor. Tab 1, Key regulators in donor 1; Tab 2, Key regulators in donor 2; Tab 3, Key regulators in donor 3; Tab 4, Key regulators in donor 4.
