# Supplementary material for: Osteogenic Differentiation Potential of Mesenchymal Stem Cells Using Single Cell Multiomic Analysis
Source: Genes (Basel). 2023 Sep 26;14(10):1871. doi: 10.3390/genes14101871 (PMC10606235; doi:10.3390/genes14101871)
Supplement: Supplementary file 1 [file genes-14-01871-s001.zip › Table S1.pdf]

Table S1. Donor’s age and sex information.

Donor’s number	Donor’s age	Donor’s gender
1	37	Male
2	36	Male
3	25	Male
4	24	Female
