# Supplementary figures and images for: Osteogenic Differentiation Potential of Mesenchymal Stem Cells Using Single Cell Multiomic Analysis
Source: Genes (Basel). 2023 Sep 26;14(10):1871. doi: 10.3390/genes14101871 (PMC10606235; doi:10.3390/genes14101871)

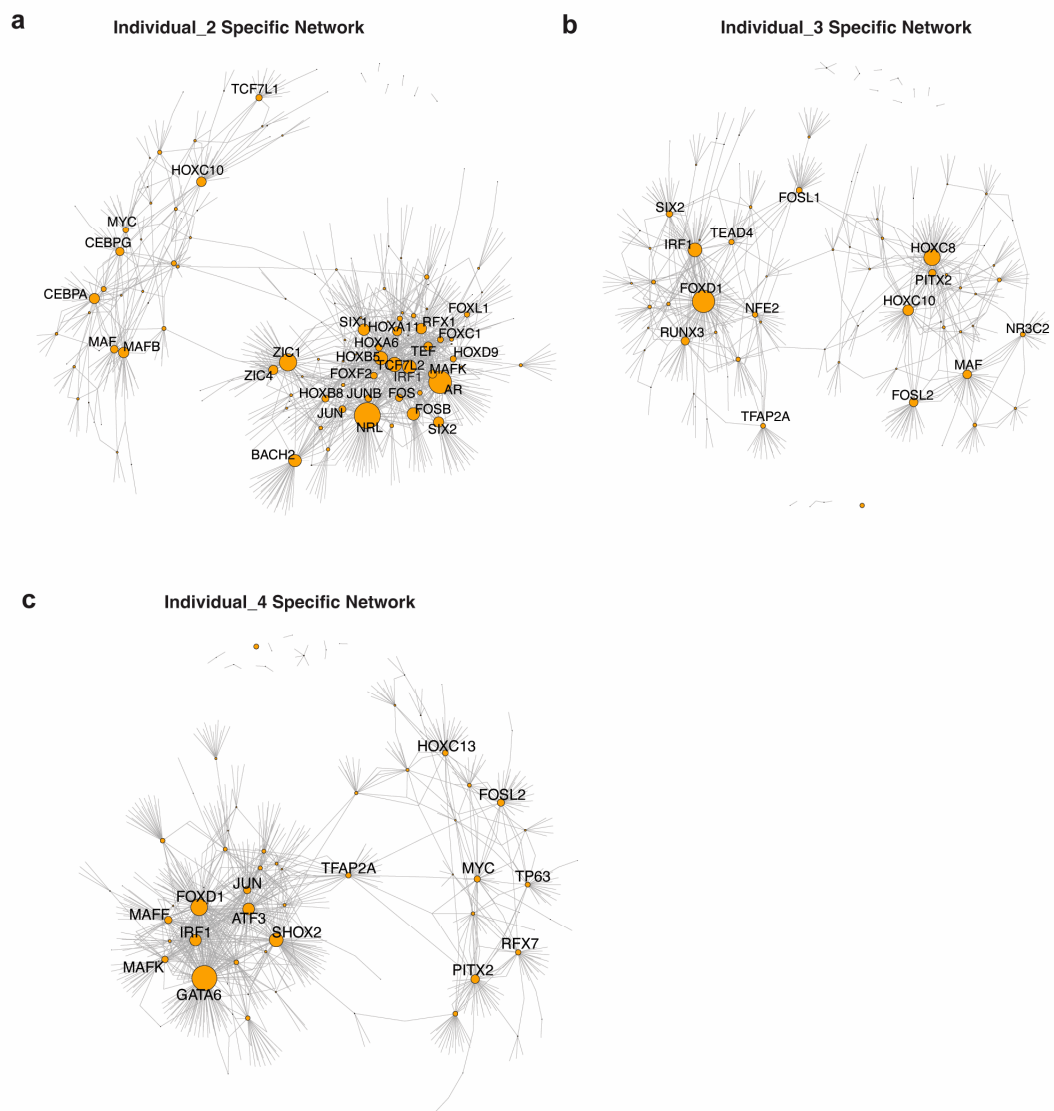

**Figure S1.**

Supplement: Supplementary file 1 [file genes-14-01871-s001.zip › Figure S1.pdf]
